# A Hybrid Interpolation Weighted Collaborative Filtering Method for Anti-cancer Drug Response Prediction

**DOI:** 10.3389/fphar.2018.01017

**Published:** 2018-09-12

**Authors:** Lin Zhang, Xing Chen, Na-Na Guan, Hui Liu, Jian-Qiang Li

**Affiliations:** ^1^School of Information and Control Engineering, China University of Mining and Technology, Xuzhou, China; ^2^College of Computer Science and Software Engineering, Shenzhen University, Shenzhen, China

**Keywords:** anti-cancer drug response, drug response prediction, recommender system, collaborative filtering, interpolation weighted method

## Abstract

Individualized therapies ask for the most effective regimen for each patient, while the patients' response may differ from each other. However, it is impossible to clinically evaluate each patient's response due to the large population. Human cell lines have harbored most of the same genetic changes found in patients' tumors, thus are widely used to help understand initial responses of drugs. Based on the more credible assumption that similar cell lines and similar drugs exhibit similar responses, we formulated drug response prediction as a recommender system problem, and then adopted a hybrid interpolation weighted collaborative filtering (HIWCF) method to predict anti-cancer drug responses of cell lines by incorporating cell line similarity and drug similarity shown from gene expression profiles, drug chemical structure as well as drug response similarity. Specifically, we estimated the baseline based on the available responses and shrunk the similarity score for each cell line pair as well as each drug pair. The similarity scores were then shrunk and weighted by the correlation coefficients drawn from the know response between each pair. Before used to find the K most similar neighbors for further prediction, they went through the case amplification strategy to emphasize high similarity and neglect low similarity. In the last step for prediction, cell line-oriented and drug-oriented collaborative filtering models were carried out, and the average of predicted values from both models was used as the final predicted sensitivity. Through 10-fold cross validation, this approach was shown to reach accurate and reproducible outcome for those missing drug sensitivities. We also found that the drug response similarity between cell lines or drugs may play important role in the prediction. Finally, we discussed the biological outcomes based on the newly predicted response values in GDSC dataset.

## Introduction

One of the top challenges in individualized therapies is the choice of the most effective chemotherapeutic regimen for each patient, while the administration of ineffective chemotherapy may increase mortality and decrease quality of life in cancer patients (Chen et al., 2013). Thus, it is urgent to evaluate each patients' possible response to each chemotherapeutic regimen to make sure the regimens applied are most likely to be effective. To address this problem, extensive patient drug screening projects need to be carried out so as to unveil significant drug response patterns. However, the large populations of cancer patients with numerous drugs has become the bottleneck.

To circumvent this issue in the context of cancer, some large drug screening projects have been carried out using cancer cell lines instead of individual cancer patients. These are NCI-60 panel, Genomics of Drug Sensitivity in Cancer (GDSC) and the Cancer Cell Line Encyclopedia (CCLE) projects (Boyd and Paull, 1995; Barretina et al., 2012; Yang et al., 2013). The NCI-60 study was pioneered by the US National Cancer Institute (NCI) to assemble the NCI60 tumor cell line panel, which has been assayed for its sensitivity to over 130,000 compounds and had been extensively profiled at the biological level (Shoemaker, 2006). It has been useful for the development of computational approaches aiming at linking drug sensitivity with genotype profiles together (Shoemaker et al., 1988; Weinstein et al., 1997; Garnett et al., 2012). The GDSC project is, to date, the largest public resource for information on drug sensitivity in human cancer cell lines and molecular markers of drug response. It pioneered the combination of drug and cell line information, including gene expression, gene copy number variations, and mutation profiles for drug sensitivity prediction (Garnett et al., 2012; Yang et al., 2013). It systematically addressed the issue of predictive biomarker identification by collectively analyzing the clinically-relevant human cell lines and their pharmacological profiles for corresponding cancer drugs. The other widely used database, CCLE (Barretina et al., 2012), collects gene expression, chromosomal copy number and massively parallel sequencing data from 947 human cancer cell lines, coupled with pharmacological profiles for 24 anti-cancer drugs across 479 of the cell lines. It allows identification of genetic, lineage, and gene expression-based predictors of drug sensitivity.

Corresponding to the large-scale datasets screened on cultured human cell line panels, many computational methods have been developed for the elucidation of the response mechanism of anti-cancer drugs, most commonly are multivariate linear regression (LASSO and elastic net regularizations) and nonlinear regression (e.g., neural networks and some kernel based methods; Barretina et al., 2012; Garnett et al., 2012; Heiser et al., 2012; Menden et al., 2013; Yang et al., 2013; Costello et al., 2014). Deamen et al. used least squares-support vector machine and random forest to identify drug response associated molecular features in breast cancer (Daemen et al., 2013). Based on the NCI-60 panel, a weighted voting classification model, an ensemble regression model using Random Forest as well as a simultaneous machine learning modeling of chemical and cell line information have been developed to predict anti-cancer drug sensitivity (Staunton et al., 2001; Riddick et al., 2011; Cortes-Ciriano et al., 2016). Based on the GDSC dataset, Ammad-uddin et al. developed a kernelized Bayesian matrix factorization (KBMF) method to integrate genomic and chemical properties as well as drug target information for drug sensitivity prediction (Ammad-ud-din et al., 2014). Sheng et al predicted unseen drug responses by calculating a weighted average of observed drug responses based on drug specific cell line similarity and drug structure similarity (Breese et al., 1998). Liu et al. proposed a dual-layer cell line drug integrated network (DLN) model, which integrated both cell line and drug similarity network data, to predict the missing drug response (Zhang et al., 2015). Wang et al. proposed HNMDRP method, incorporating gene expression, chemical structure as well as drug target and protein-protein interaction information to predict missing values of drug responses in cell lines (Zhang et al., 2018). Based on the transcriptomic data from both GDSC and CCLE, Kim et al. developed a network-based classifier for predicting sensitivity of cell lines to anti-cancer drugs (Kim et al., 2016). Base on the same whole datasets, Wang et al. proposed a similarity-regularized matrix factorization (SRMF) method for drug response prediction, which incorporates similarities of drugs and of cell lines simultaneously (Wang et al., 2017). Stanfield et al. proposed a heterogeneous network based method to predict the interaction between cell line-drug pairs (Stanfield et al., 2017). They classified the interaction between each cell line-drug pairs into sensitive and resistant, thus, turned the prediction problem into classification. Current methods have taken the similarity of genomic or transcriptomic profiles as well as drug structure into consideration for similarity definition, which were often defined by calculating the Pearson correlation coefficient for genomic profiles, or Jaccard coefficient for drug chemical fingerprint in present studies and are called as *COEF* in the following for short. However, the similarity that exhibited through drug sensitivity, which can be defined by calculating the Pearson correlation coefficient based on drug response sensitivity, has not been considered yet and is called as *RPCC* for short in the following. Not to mention the combination of *COEF* and *RPCC*, which is called as *MRPCC* (Multiplication of *COEF* and *RPCC*) for short throughout the paper. Drug-target interaction and PPI network have also been considered to improve the prediction performance (Chen et al., 2012; Stanfield et al., 2017).

Regarding the relatively more credible assumption that similar cell lines and similar drugs exhibit similar drug responses (Zhang et al., 2015), the prediction of missing drug response can be considered as a typical Recommender System (RS) (Adomavicius and Tuzhilin, 2005). Typically, in a recommender system, there is a set of users and a set of items. Each user rates a set of items by some values. The recommender system attempts to profile user preferences and tries to model the interaction between users and items, which is exactly what we want in the issue of drug response prediction. The cell lines correspond to users while drugs correspond to items. From the RS perspective, the similarity shown through drug sensitivity is also very important for missing value prediction. Thus, we improved an RS technique, Hybrid Interpolation Weighted Collaborative Filtering (HIWCF) (The acronym list defined in this paper is shown in Table 1), for drug response prediction, which incorporates similarities of drugs and of cell lines in additional to the known drug response simultaneously (The key source code and ready to use CCLE and GDSC datasets are provided at https://github.com/laureniezhang/HIWCF). To demonstrate its effectiveness, we compared HIWCF with SRMF and KBMF, which have been proved to show higher performance than typical similarity-based methods. The evaluation metrics used were averaged Pearson correlation coefficient (PCC) and averaged root mean square error (RMSE) over all drugs. The results on GDSC and CCLE drug response datasets by 10-fold cross validation showed that similarity defined based on drug response is more dependable for unknown response prediction, and the incorporation of gene expression profile, drug response, and drug structure similarity help to better improve the prediction performance. Finally, HIWCF was applied to impute the unknown drug response values in GDSC dataset for further evaluation.

**Table 1:** Acronym list.

**Acronym**	**Detailed description**
HIWCF	Hybrid Interpolation Weighted Collaborative Filtering
*COEF_*c*_*	Pearson Correlation Coefficient drawn from cell line gene expression profile
*COEF_*d*_*	Jaccard Correlation Coefficient drawn from drug chemical fingerprint
*RPCC_*c*_*	Pearson Correlation Coefficient between cell lines drawn from drug response matrix
*RPCC_*d*_*	Pearson Correlation Coefficient between drugs drawn from drug response matrix
*RPCC*	Refers to *RPCC_*c*_* or *RPCC_*d*_*. It depends on the context.
*MRPCC_*c*_*	Multiplication of *COEF_*c*_* with *RPCC_*c*_*, used as final similarity score between cell lines.
*MRPCC_*d*_*	Multiplication of *COEF_*d*_* with *RPCC_*d*_*, used as final similarity score between drugs.
*MRPCC*	Refers to *MRPCC_*c*_* or *MRPCC_*d*_*. It depends on the context.

## Materials and methods

### Data and preprocessing

In this paper, two datasets, both consisting of large scale genomic expression profiles, pharmacologic profiling of drug compounds, as well as the experimentally determined drug response measurements IC50 values (the concentration of a drug compound that reached the absolute inhibition of 50% *in vitro*, given as natural log of μM) or experimental activity areas were used for performance evaluation. Large scale genomic expression profiles were normalized across cell lines to draw the similarity matrix of cell lines. The chemical structures of drug compounds were used to draw the similarity matrix of drugs.

The first dataset is from GDSC project (http://www.cancerrxgene.org/), consisting of 139 drugs and a panel of 790 cancer cell lines (release 5.0). We selected 652 cell lines for which both drug response data and gene expression were available, and 135 drugs whose SDF format (encoding the chemical structure of the drugs) were available. The drug response is given with IC50 values (70,676 data points, matrix 80.3% complete).

The second dataset consists of 1,036 human cancer cell lines and 24 drugs, which is from CCLE project (http://www.broadinstitute.org/ccle). We also selected 491 cell lines and 23 drugs following the same rule used in GDSC dataset. The drug response is given with activity areas (10,870 data points, matrix 96.25% complete). Both ready to use datasets are submitted to Github at https://github.com/laureniezhang/HIWCF.

### Problem formulation

We basically treat anti-cancer drug response prediction as a RS problem where each cell line-drug pair is the typical user-item pair. Based on the finding that similar cell lines by gene expression profiles exhibit similar response to the same drug (Zhang et al., 2015), we proposed a weighted interpolation collaborative filtering method to approximate the sensitivity of cell line *u* to drug *i*. For convenience, we reserve special indexing letters for distinguishing cell lines from items: for cell lines *u, v*, and for drugs *i, j*. We are given cell line drug response about *m* cell lines and *n* drugs, arranged as an *m* × *n* matrix *R* = {*r*_*ui*_}_1≤*u*≤*m*,1≤*i*≤*n*_, where higher value of activity area or lower value of IC50 means a better sensitivity of a cell line to a given drug.

### Baseline estimate strategy

Since typical CF data often exhibit large user and item effects, that means systematic tendencies for some users to give higher ratings than others, and for some items to receive higher ratings than others, we first adjusted the rating data by accounting for these effects, which we include in the baseline estimate strategy. Let μ denotes the overall average drug response, we denote the estimated baseline for an unknown rating ${\hat{r}}_{ui}$ as *b*_*ui*_, which accounts for the above-mentioned user and item effects.

(1) $$\begin{array}{l} b_{ui}=\mu +b_{u}+b_{i} \end{array}$$

The parameters *b*_*u*_ and *b*_*i*_ indicate the observed deviations of cell line *u* and drug *i*, respectively, from the average.

In order to get the baseline formulation, for each drug *i*, we set:

(2) $$\begin{array}{l} b_{u}=\frac{\sum_{i\in U\left(u,i\right)}\left(r_{ui}-\mu -b_{i}\right)}{\lambda_{3}+|U\left(u,i\right)|} \end{array}$$

Then, for each cell line *u*, we set:

(3) $$\begin{array}{l} b_{i}=\frac{\sum_{u\in U\left(u,i\right)}\left(r_{ui}-\mu \right)}{\lambda_{2}+|U\left(u,i\right)|} \end{array}$$

where *U*(*u, i*) is the set of cell lines who responses to drug *i*, or the set of drugs who have responses in cell line *u*, and |*U*(*u, i*)| means the number of elements in set*U*(*u, i*). λ_2_and λ_3_ are regularization parameters that help to shrink the averages *b*_*u*_ and *b*_*i*_ toward zero. They are set to 5 and 2, respectively in the following simulation process.

### Similarity definition

The similarity matrixes are required for identification of K nearest neighbors. The original similarity of cell lines was drawn based on the Pearson correlation coefficient between the gene expression profiles of cell line *u* and *v*, which is indicated as *COEF*_*c*_*uv*__. The *c* in the subscript refers to cell line-oriented. The similarity of drugs was drawn based on the Jaccard coefficient between the drug chemical structures of drug *i* and *j*, which is indicated as *COEF*_*d*_*ij*__. The *d* in the subscript refers to drug-oriented.

However, to some extent, the similarity between cell line *u* and *v* can also be shown from their drug response. Thus, in this paper, we investigated the performance of different similarity definitions for drug response prediction. To be more specific, the similarity of cell line *u* and *v*, indicated as*MRPCC*_*c*_*uv*__, was defined as the multiplication of *COEF*_*c*_*uv*__and*RPCC*_*c*_*uv*__, which helps the cell line pairs with consistent similarity in gene expression and drug response to get higher rank for unknown response prediction.

(4) $$\begin{array}{l} MRPCC_{c_{uv}}\leftarrow COEF_{c_{uv}}\times RPCC_{c_{uv}} \end{array}$$

where *COEF*_*c*_*uv*__ was defined as the their gene expression profile's Pearson correlation, while *RPCC*_*c*_*uv*__ was defined as the correlation between the response IC50 value of cell line *u* and *v*.

(5) $$\begin{array}{l} RPCC_{c_{uv}}=\frac{\sum \left(R_{u•}-{\bar{R}}_{u•}\right)\left(R_{v•}-{\bar{R}}_{v•}\right)}{\sqrt{\sum {\left(R_{u•}-{\bar{R}}_{u•}\right)}^{2}\sum {\left(R_{v•}-{\bar{R}}_{v•}\right)}^{2}}} \end{array}$$

where *R*_*u*•_ represents the response value of the *u*-th cell line, and ${\bar{R}}_{u•}$ represents the mean of the *u*-th cell line's response.

In the same way, the similarity between drug *i* and *j*, indicated as *MRPCC*_*d*_*ij*__, was defined as the multiplication of *COEF*_*d*_*ij*__ and *RPCC*_*d*_*ij*__.

(6) $$\begin{array}{l} MRPCC_{d_{ij}}=COEF_{d_{ij}}\times RPCC_{d_{ij}} \end{array}$$

where *COEF*_*d*_*ij*__ was defined as their drug chemical fingerprint's Jaccard coefficient, while *RPCC*_*d*_*ij*__ was defined as the Pearson correlation coefficient between response IC50 values of drug *i* and *j*.

(7) $$\begin{array}{l} RPCC_{d_{ij}}=\frac{\sum \left(R_{•i}-{\bar{R}}_{•i}\right)\left(R_{•j}-{\bar{R}}_{•j}\right)}{\sqrt{\sum {\left(R_{•i}-{\bar{R}}_{•i}\right)}^{2}\sum {\left(R_{•j}-{\bar{R}}_{•j}\right)}^{2}}} \end{array}$$

where *R*_•*i*_ represents the response value of the *i*-th drug, and ${\bar{R}}_{•i}$ represents the mean of the *i*-th drug's response.

In order to avoid the bias caused by the different level of support (different number of known responses) for each cell line-drug pair, we also went through a shrunk procedure for similarity score, which is denoted by (Koren, 2010):

(8) $$\begin{array}{l} w_{i,j}\leftarrow \frac{\left|U\left(i,j\right)\right|}{|U\left(i,j\right)|+\lambda_{4}}w_{i,j} \end{array}$$

where |*U*(*i, j*)| is the number of cell lines who have responses to both drug *i* and *j*, or the number of drugs who have responses from both cell line *i* and *j*. *w*_*ij*_ is the similarity *MRPCC*_*c*_ defined in (4) and *MRPCC*_*d*_ in (6). λ_4_is a constant, which is set as 50 in the experiments.

In the following, we adopted a case amplification strategy, which refers to a transform applied to the weights used in the following collaborative filtering prediction, to reduce the noise in the data. The transform emphasizes high weights and punishes low weights by (Breese et al., 1998):

(9) $$\begin{array}{l} w_{i,j}\leftarrow w_{i,j}▪|w_{i,j}{|}^{\rho -1} \end{array}$$

where ρ is the case amplification power, ρ ≥ 1, and we also followed the typical choice of ρ as 2.5 (Lemire, 2005).

### Drug response prediction based on HIWCF method

After removing the noise by baseline estimate strategy, we need to predict the unknown sensitivity for cell line *u* of drug *i*, which is ${\hat{r}}_{ui}$. Based on the above-mentioned similarity measure *w* defined in (9), we first conducted drug-oriented CF, and *k* drugs, which are most similar to drug *i* that had responses in cell line *u* were identified. This set of *k* neighboring drugs is denoted by *U*(*i*; *u*). Then, based on *w*, we conducted cell line-oriented CF, and *k* cell lines that responded to drug *i*, which are most similar to cell line *u* were identified. This set of *k* neighboring cell lines is denoted by *U*(*u*; *i*). Finally, the predicted value of ${\hat{r}}_{ui}$ is taken as an average of the weighted average of the response of neighboring drugs found in *U*(*i*; *u*) and that of the response of neighboring cell lines found in*U*(*u*; *i*), while adjusting from user and item effects through baseline estimates:

(10) $$\begin{array}{l} {\hat{r}}_{ui}=b_{ui}+\frac{1}{2}(\frac{\sum_{j\in U(i;u)}w_{i,j}(r_{uj}-b_{uj})}{\sum_{j\in U(i;u)}w_{i,j}}\\ \;+\frac{\sum_{v\in U(u;i)}w_{i,j}(r_{vi}-b_{vi})}{\sum_{v\in U(u;i)}w_{i,j}}) \end{array}$$

## Results

### Similarity exhibited in drug response sensitivity shows leading role in prediction

We first conducted 10-fold cross validation to evaluate the performance of different similarity definition. Incorporated with *COEF, RPCC* as well as *MRPCC*, drug response prediction performance of HIWCF is evaluated in both CCLE dataset and GDSC dataset with activity area or IC50 value as drug response measurement in comparison with KBMF and SRMF. The evaluation measures included average PCC, RMSE between predicted and observed drug responses through all drugs. Considering the known fact that the sensitive and resistant cell lines of each drug are more valuable to unveil mechanisms of drug actions, we also included PCC and RMSE from sensitive and resistant cell lines for each drug, which were denoted as PCC_S/R and RMSE_S/R (Wang et al., 2017).

For each dataset, the drug response entries were divided into 10-folds randomly with almost the same size. Each time, one-fold was used as the test set, while the rest nine-folds were used as the training set. The prediction was repeated 10 times such that each fold acted as a test set once. The whole cross-validation was run for 100 times for each dataset, and the prediction performance was shown in Tables 2, 3.

**Table 2:** The comparison results between HIWCF with different similarity definition (MRPCC/RPCC/COEF), SRMF, and KBMF obtained under 10-fold cross validation on CCLE dataset.

**Methods**		**Drug-averaged PCC_S/R**	**Drug-averaged RMSE_S/R**	**Drug-averaged PCC**	**Drug-averaged RMSE**
HIWCF	MRPCC	0.80(±0.07)	0.66(±0.21)	0.74(±0.08)	0.53(±0.15)
	RPCC	0.80(±0.06)	0.67(±0.22)	0.73(±0.08)	0.54(±0.16)
	COEF	0.74(±0.06)	0.76(±0.27)	0.66(±0.06)	0.60(±0.20)
SRMF		0.78(±0.07)	0.74(±0.23)	0.71(±0.09)	0.57(±0.18)
KBMF		0.65(±0.10)	0.81(±0.20)	0.71(±0.10)	0.64(±0.17)

**Table 3:** The comparison results between HIWCF with different similarity definition (MRPCC/RPCC/COEF), SRMF, and KBMF obtained under 10-fold cross validation on GDSC dataset.

**Methods**		**Drug-averaged PCC_S/R**	**Drug-averaged RMSE_S/R**	**Drug-averaged PCC**	**Drug-averaged RMSE**
HIWCF	MRPCC	0.68(±0.14)	1.88(±0.54)	0.58(±0.15)	1.51(±0.39)
	RPCC	0.68(±0.14)	1.87(±0.53)	0.58(±0.15)	1.50(±0.38)
	COEF	0.57(±0.15)	2.12(±0.60)	0.46(±0.14)	1.66(±0.43)
SRMF		0.71(±0.15)	1.73(±0.46)	0.62(±0.16)	1.43(±0.36)
KBMF		0.59(±0.14)	2.00(±0.51)	0.49(±0.14)	1.59(±0.42)

As is shown, the prediction performance of HIWCF with *MRPCC/RPCC* similarity were far better than that with *COEF* similarity, which suggested that the similarity exhibited in drug response may lead important role than that of gene expression profiles or drug structures in the scenario of drug response prediction. Thus, we turned to use the predicted values of HIWCF with *MRPCC* similarity measure only in the rest evaluation of our paper.

In Table 2, we can also see that in CCLE dataset, the performance of HIWCF with *RPCC* and *MRPCC* were better than that of SRMF, without mentioning KBMF. However, as shown in Table 3, the performance of HIWCF with either *RPCC* or *MRPCC* were a little bit worse than that of SRMF. That may be because the similarity score of *RPCC/MRPCC* is based on the known drug response for each cell line-drug pair. Since GDSC dataset is much sparser than that of CCLE, the similarity score of *RPCC/MRPCC* of GDSC is less reliable than that of CCLE.

**Figure 1:**
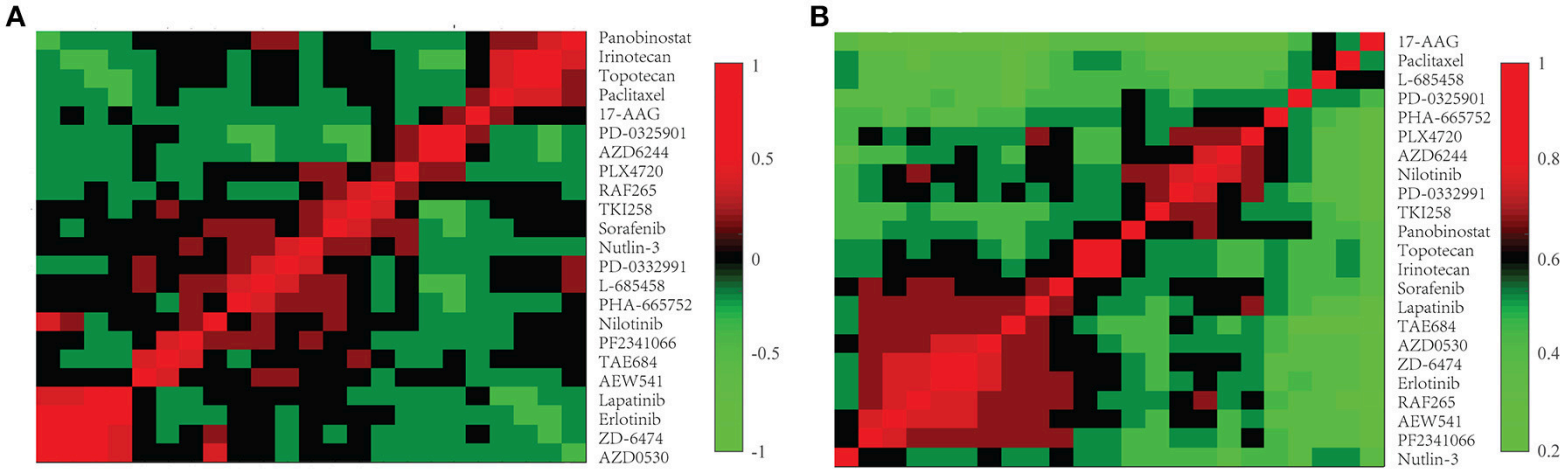
The drug similarity *RPCC* and *COEF* of 23 drugs in CCLE dataset. **(A)** The plot shows *RPCC* similarity for 23 drugs in CCLE dataset. **(B)** The plot shows *COEF* similarity for 23 drugs in CCLE dataset.

**Figure 2:**
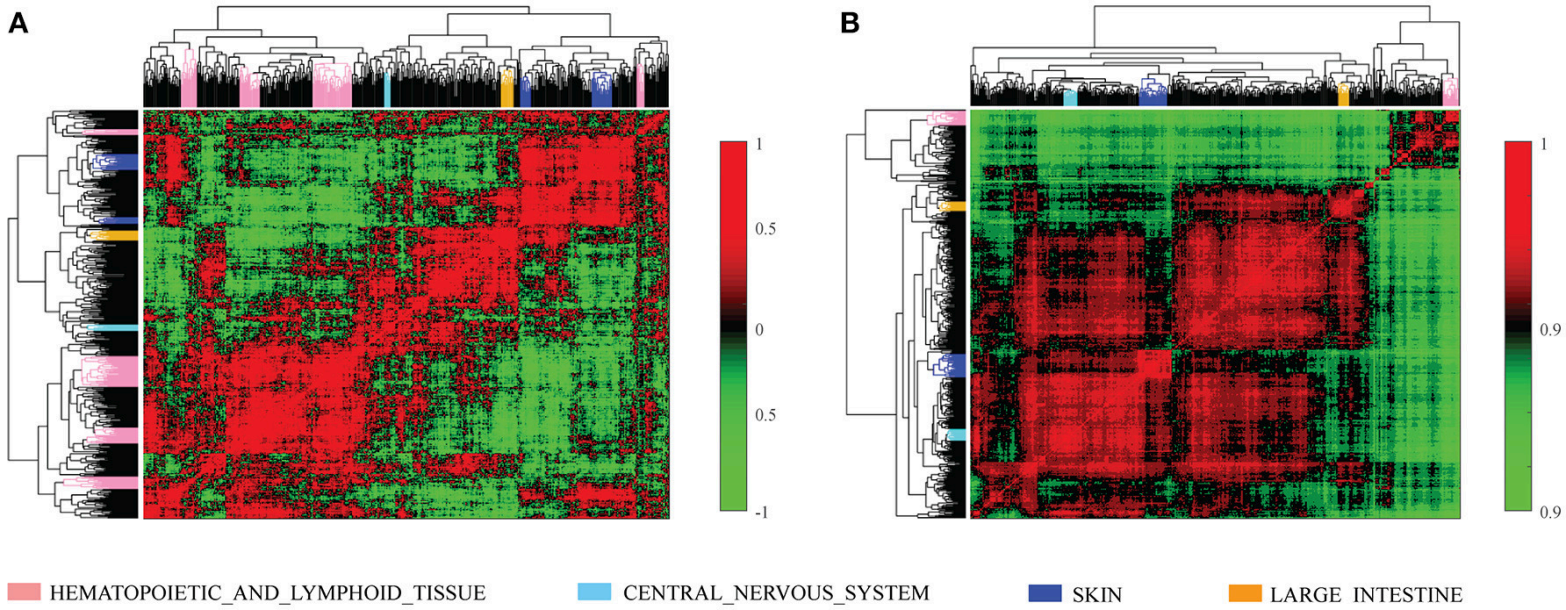
The cell line similarity *RPCC* and *COEF* of 491 cell lines in CCLE dataset. **(A)** The plot shows *RPCC* similarity for 491 cell lines in CCLE dataset. **(B)** The plot shows *COEF* similarity for 491 cell lines in CCLE dataset.

**Figure 3:**
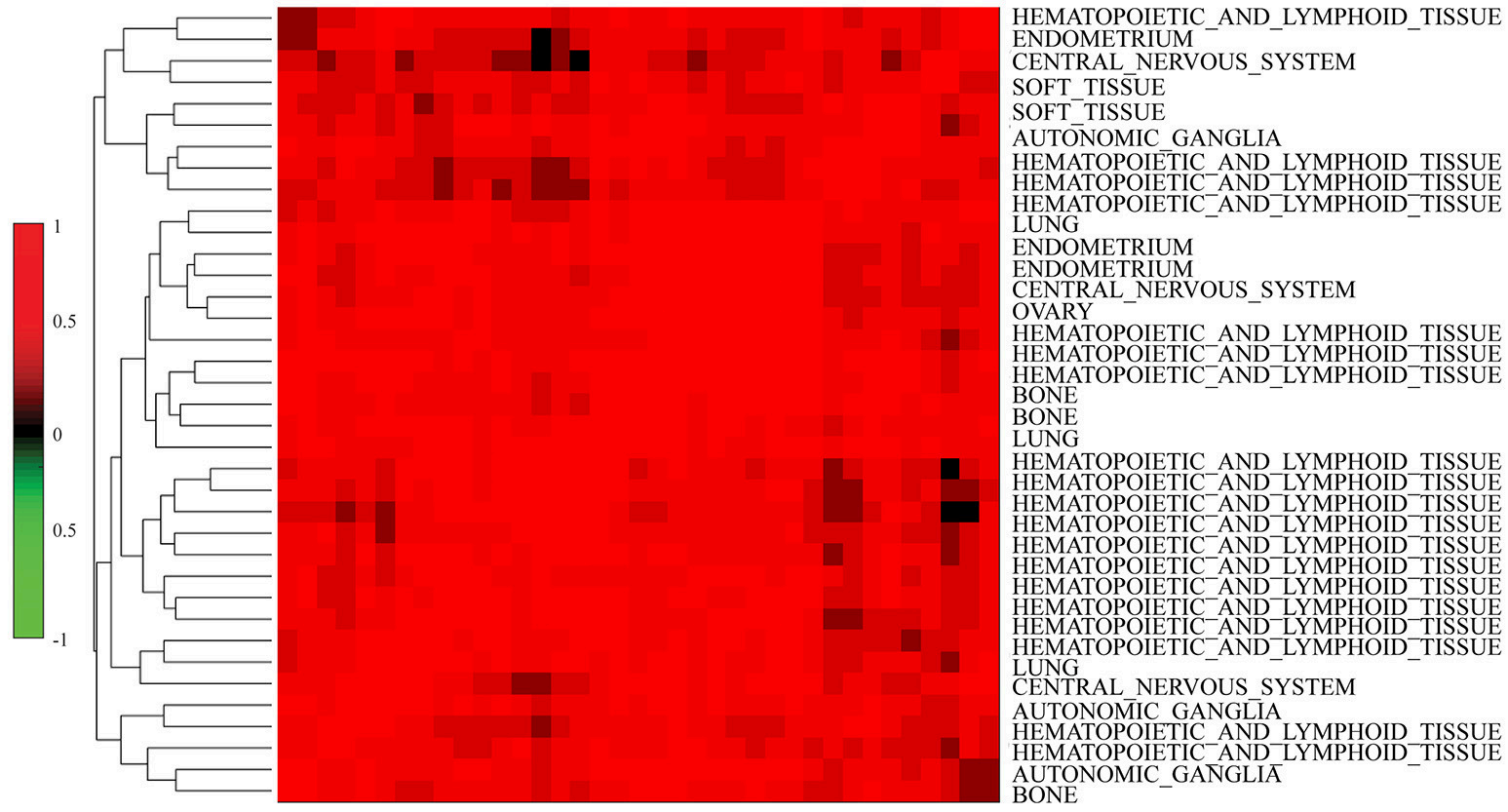
Similar cell lines are more likely to be clustered into the same group (have similar similarity score) based on *MRPCC* similarity score. Most cell lines in the plot were collected from hematopoietic and lymphoid tissues.

We further investigated the difference between *COEF* and *RPCC*. To be more specific, based CCLE dataset, we calculated the drug structure fingerprint similarity *COEF* for hierarchical clustering analysis. As shown in Figure 1B, it was surprising that the similarity score for most drug pairs were approaching 1, which was undistinguishable for neighbor selection. However, we can get distinguishable similarity scores from drug response similarity *RPCC*, as shown in Figure 1A. If we investigate the drugs that clustered into the same group, such as “Lapatinib,” “AZD0530,” “ZD-6474,” and “Erlotinib.” It is well-known that they are EGFR inhibitors, thus, they are most likely have higher similarity scores in drug response (Yuan et al., 2016). We also investigate the gene expression similarity with cell line response similarity. The cell line response similarity *RPCC* and cell line gene expression similarity *COEF* were calculated for hierarchical clustering, which were comparable with each other (Figure 2). The results show that cell lines collected from the same tissue type may have higher similarity score, which is consistent with previous studies. For example, most cell lines that clustered into the same group shown in Figure 3 were collected from hematopoietic and lymphoid tissues. Hierarchical clustering was achieved in both row and column direction, with original similarity score was normalized with 0 mean.

### Cross-validation on CCLE drug response datasets

We then tested the prediction performance of HIWCF for 23 drugs tested in the CCLE study, which were quantified based on PPC and RMSE between the predicted and observed activity areas.

**Figure 4:**
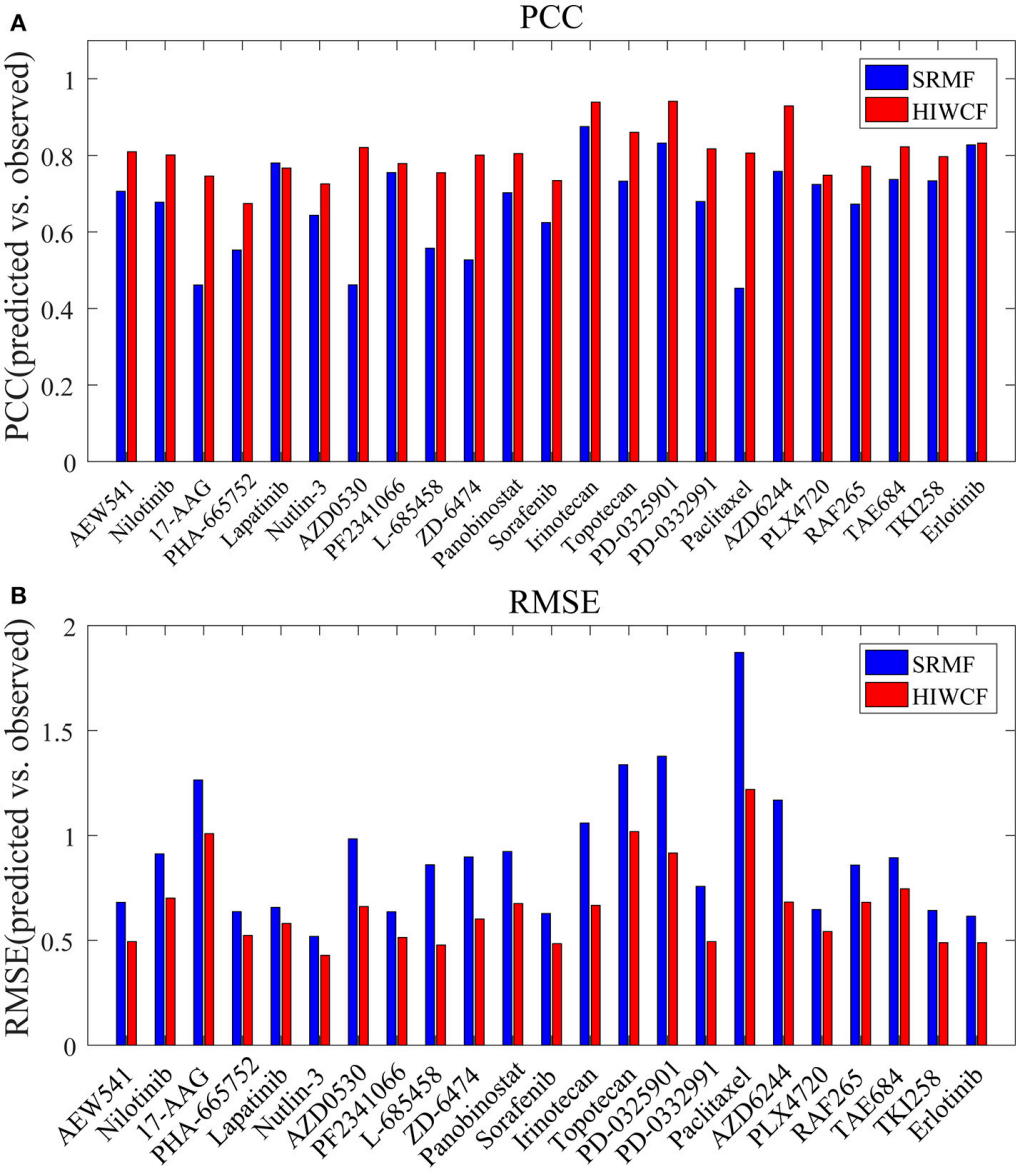
Prediction performance of HIWCF with *MRPCC* similarity and SRMF for all 23 drugs tested in the CCLE dataset. **(A)** Bar plot shows that the prediction performance of HIWCF with *MRPCC* is better than that of SRMF in the perspective of Pearson correlations between the predicted and observed activity areas. **(B)** Bar plot shows that the prediction performance of HIWCF with *MRPCC* is better than that of SRMF in the perspective of Root Mean Square Error between the predicted and observed activity areas.

**Figure 5:**
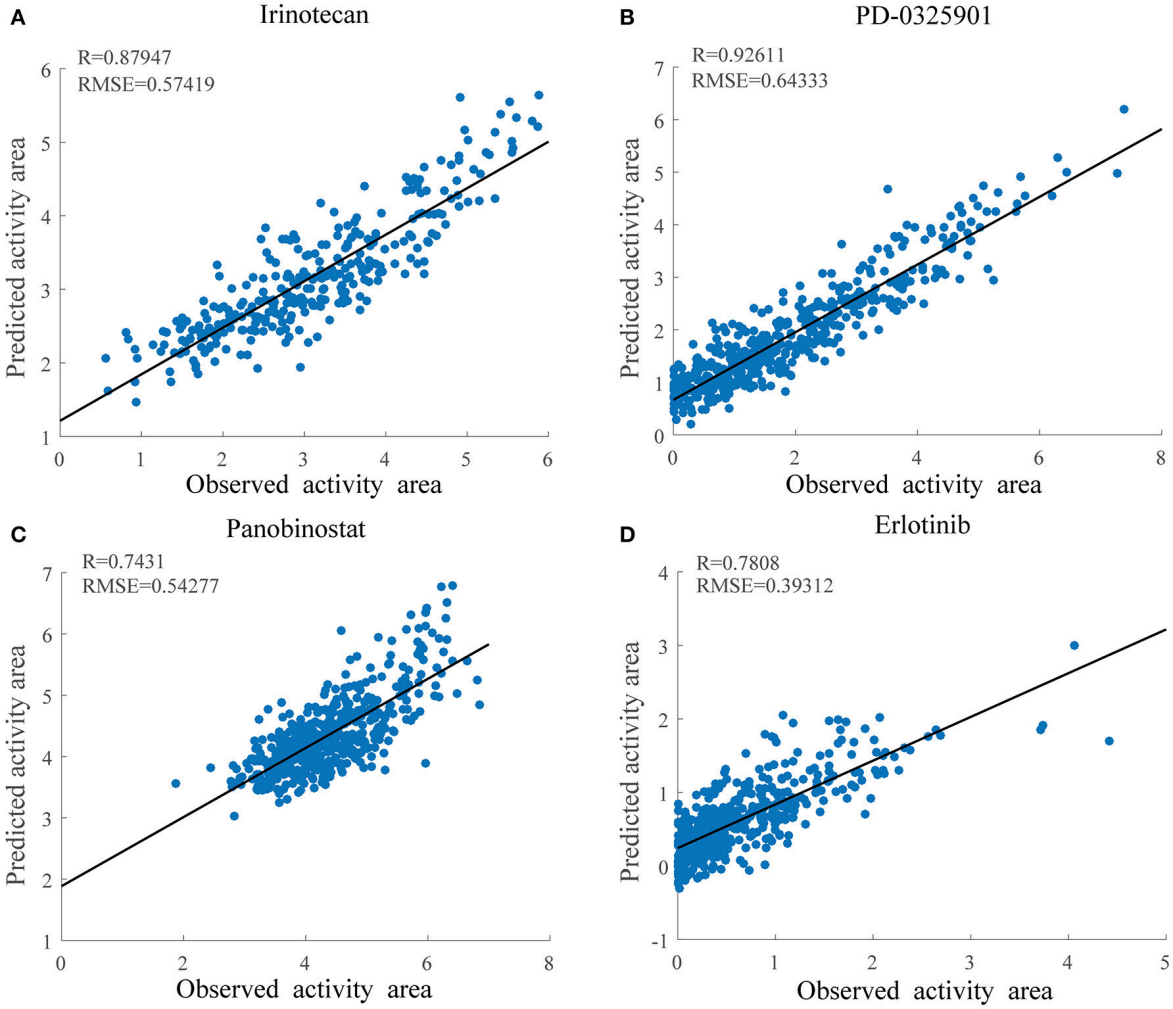
Scatter plots of observed and predicted drug activity area for four drugs in CCLE using HIWCF with MRPCC similarity. **(A)** Scatter plot of Irinotecan. **(B)** Scatter plot of PD-0325901. **(C)** Scatter plot of Panobinostat. **(D)** Scatter plot of Erlotinib.

As shown in Figure 4, the overall prediction performance of HIWCF throughout all the drugs was significantly higher than that of SRMF for the CCLE dataset. We believe that the improvement of HIWCF is most likely due to the involvement of similarity calculated from response matrix. The scatter plots of observed vs. predicted responses for four demonstrative drugs, Irinotecan, PD-0325901, Panobinostat, and Erlotinib are shown in Figure 5, which indicate the good correlations between existing response and predicted ones.

### Response data prediction in GDSC data

**Figure 6:**
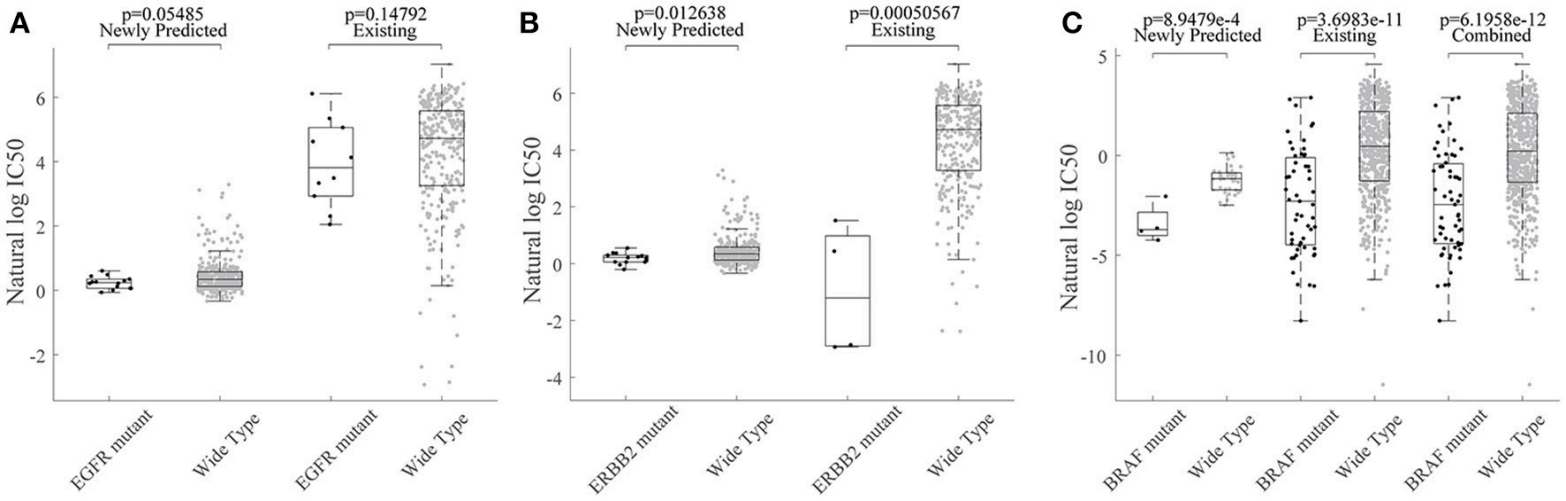
The association of lapatinib sensitivity and cancer gene mutations were consistent for predicted response values. WT refers to the non-mutated (wild type) cell lines. **(A)** Box plot for grouped cell line response values for lapatinib based on their EGFR mutation profiles. **(B)** Box plot for grouped cell line response values for lapatinib based on their ERBB2 mutation profiles. **(C)** Box plot for grouped cell line response values for lapatinib based on their ERBB2 mutation profiles.

Based on the HIWCF method validated, we based on all known data to predict the unknown ones in the GDSC dataset. As in Wang et al. (2017), we also focused on an EGFR and ERBB2 inhibitor drug lapatinib, where more than half of response values (342/652) were unknown. Previous studies had demonstrated that EGFR and ERBB2 amplification was associated with sensitivity to lapatinib, which has been licensed for the treatment of HER2+ breast cancer clinically (Petrelli et al., 2017; Zhao et al., 2017). Thus, we tried to investigate whether the observed and predicted response of EGFR/ERBB2 mutated cell lines exhibit the sensitivity to lapatinib. All the 635 cell lines in GDSC were first grouped into mutated vs. wildtype by the total copy number variation in the exact gene (Garnett et al., 2012). Then, we found that not only EGFR mutated but also ERBB2 mutated cell lines were both significantly more sensitive to lapatinib, as shown in Figures 6A,B, which was consistent with previously mentioned conclusions.

We further investigated whether the newly predicted drug responses combined with known drug responses were able to detect novel drug-cancer gene association or not. To be more specific, the oncogene BRAF has been found to be significantly associated with enhanced and selective sensitivity to MEK inhibitor PD-0325901 (Solit et al., 2006) (*p* = 3.70e-11 for known drug responses; *p* = 6.20e-12 for combined response of predicted ones and known ones; Figure 6C).

**Figure 7:**
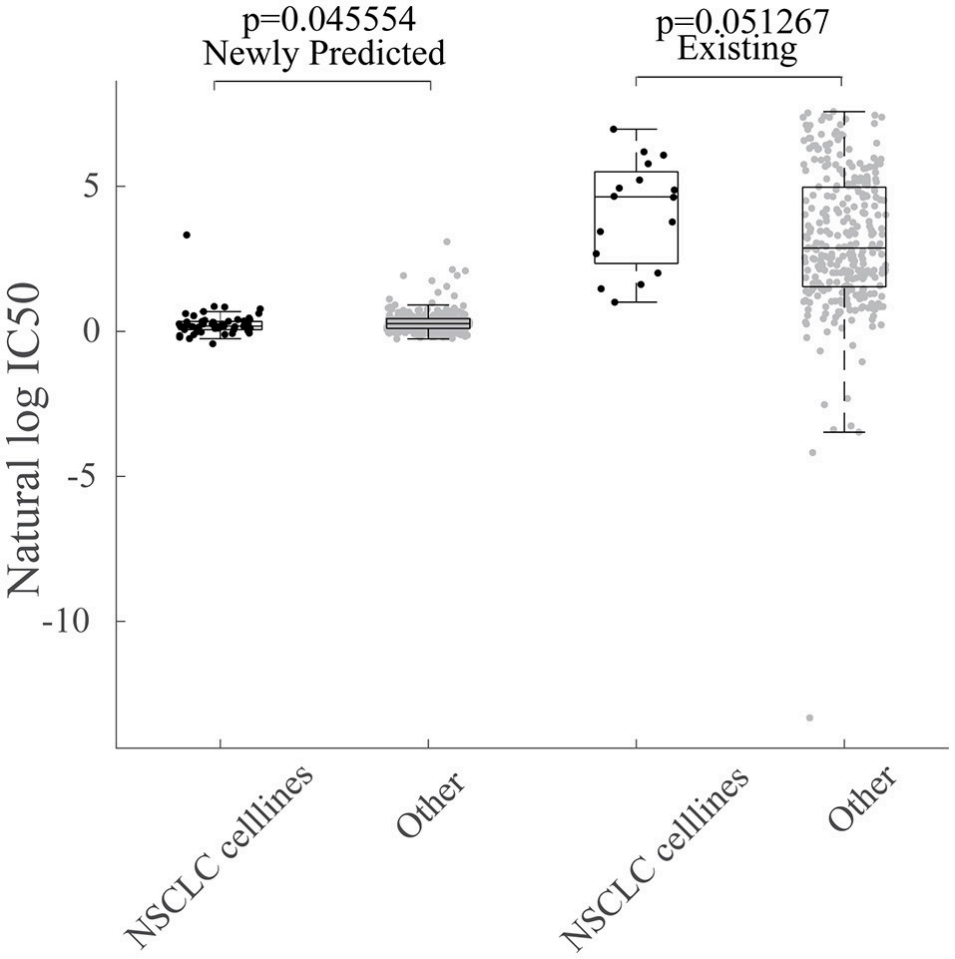
Repositioning of sunitinib. Box plot for grouped cell line response values for Sunitinib based on their tissue type. NSCLC indicates cell lines sampled from non-small cell lung cancer tissues.

The newly predicted drug responses of GDSC dataset may also aid in drug repositioning. For example, Sunitinib, as a kinase inhibitor targeting VEGFR2 and PDGFRβ, has been observed to be sensitive to non-small cell lung cancer (NSCLC) based on newly predicted drug responses vs. available ones, as shown in Figure 7.

**Figure 8:**
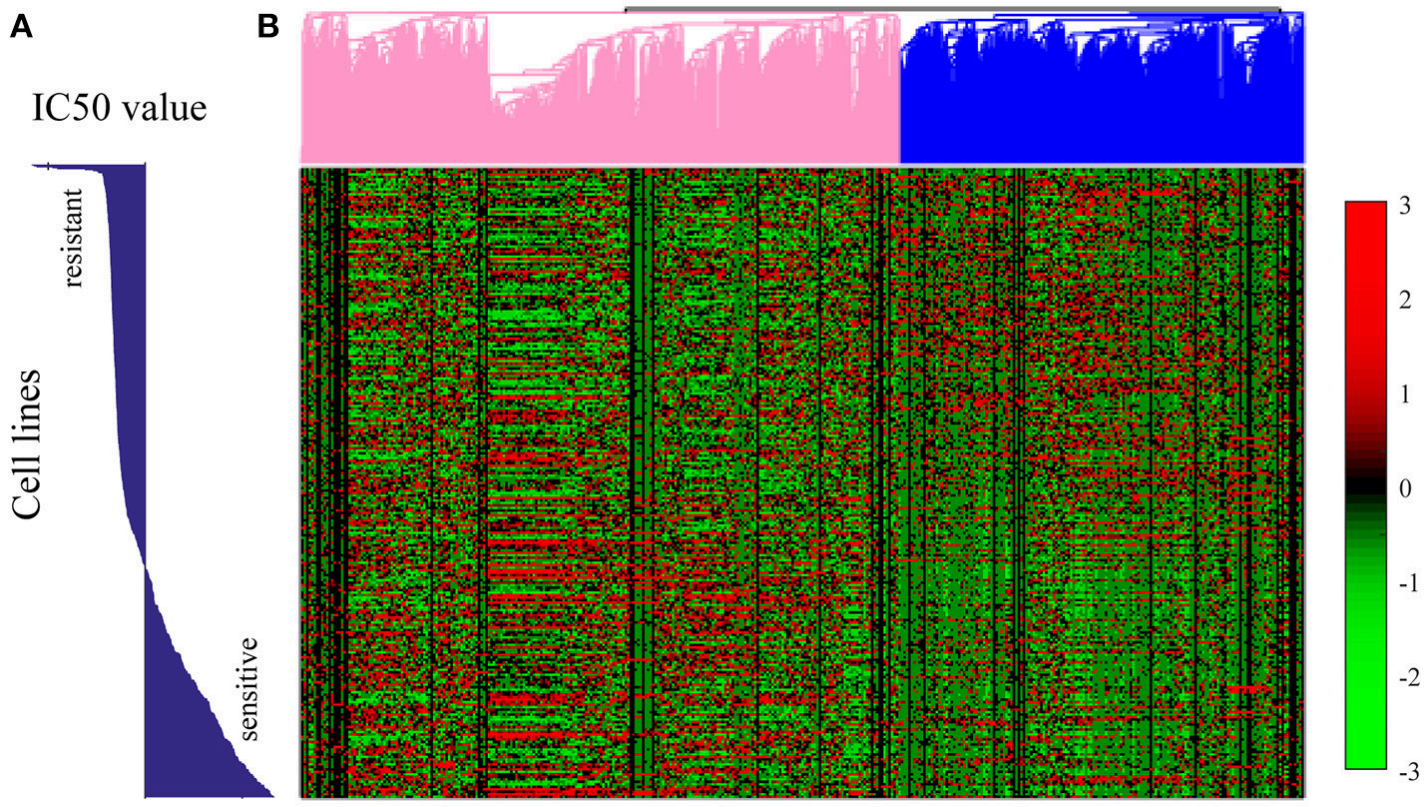
Hierarchical clustering analysis on the gene expression profiles for all the 652 cell lines in GDSC dataset. **(A)** The bar plot of the IC^50^ values of each cell line. **(B)** The hierarchical clustering plot on the right showed the gene expression pattern for 20% most variant genes in each cell line. Each row in **(A)** corresponds to the exact row in the hierarchical clustering plot of gene expression profiles in **(B)**. The genome expression pattern was shown as some genes were up-regulated in Sunitinib resistant cell lines but down-regulated in Sunitinib sensitive cell lines, while some other genes were up-regulated in Sunitinib sensitive cell lines but down-regulated in Sunitinib resistant cell lines.

We further conducted the hierarchical clustering analysis through genes based on the expression profile of all the 652 cell lines. Before hierarchical clustering, 80 percent genes that show less variations over all the genes were filtered out. As shown in Figure 8, the patterns of gene expression were shown to be related with the sensitivity of each cell line to Sunitinib. The pink marked group of genes showed higher expression in cell lines which were sensitive to Sunitinib, while the blue marked group of genes showed higher expression in cell lines which were resistant to Sunitinib.

We further conducted GO enrichment analysis for both groups of genes. For the genes that up-regulated in Sunitinib resistant cell lines were found to be related to some repair pathways, such as regulation of DNA repair (*p* = 1.1e-3), base-excision repair (*p* = 0.032), nucleotide-excision repair (*p* = 6e-3), interstrand cross-link repair (*p* = 0.01), mismatch repair (*p* = 0.048), etc., which were found to be important factors of drug resistance. For genes that were up-regulated in Sunitinib sensitive cell lines were found to be related to mTOR signaling pathway (*p* = 1e-2), NF-kappaB signaling (*p* = 4.1e-10). The inhibition of the signaling pathways help to increase drug sensitivities (Cai et al., 2014).

## Discussion

In this paper, we used a recommender system-based method HIWCF to predict anti-cancer drug sensitivity in GDSC and CCLE datasets respectively. The idea of the method comes from the fact that similar cell lines exhibit similar responses to the same drug, which is the exact motivation of a recommender system. This method first estimated the baseline, which helped to remove the noise in the original drug sensitivity, then shrunk the similarity measure by integration of gene expression profile, drug structure in addition to the correlation between cell lines and drugs exhibited in the drug response, which helped to weak the influence of sparseness in response matrix. Finally, it incorporated the user-orientated and item-orientated interpolation weighted collaborative filtering method to predict the unknown drug sensitivity values. Ten-fold cross validation demonstrated that the similarity drawn based on known drug response can better improve the prediction performance in comparison to the similarity drawn based on cell line gene expression profiles and drug structure only. At least, in the respective of recommender system method, it is more reliable to predict the unknown drug sensitivity based on the similarity exhibited in known drug responses. We also applied HIWCF method to predict the missing drug response values in GDSC dataset. To be more specific, we found the consistent conclusions of mutated cell lines such as EGFR/ERBB2 are more sensitive to the drug of lapatinib. We also found that the gene expression profiles showed exact pattern for Sunitinib sensitive and resistant cell lines. Genes that up-regulated in Sunitinib sensitive cell lines were subjected to repair pathways, while genes that down-regulated in Sunitinib resistant cell lines were subjected to some drug enhancement related pathways.

In comparison with existing drug response prediction methods, HIWCF follows a neighbor based collaborative filtering approach for unknown drug response prediction, which is theoretically simple and intuitive. Matrix Factorization based methods, such as SRMF model both cell lines and drugs with some latent factors for unknown drug response prediction.

However, this method has its own drawbacks. First, since HIWCF highly depends on the known drug response, the performance highly depends on the sparseness of the response matrix. The sparser the matrix is, the worse the performance it gets. Secondly, the similarity of cell lines is calculated by combining gene expression correlation coefficient and Pearson correlation coefficient exhibited in their known drug response. However, the similarity can also be improved by integrating the epigenetic, epi-transcriptomic information, etc. Furthermore, some pathway related information or other dynamic information may also help to improve the performance. Therefore, we can further work on some methods that aim in sparse issue as well as multi-omics integration one in the future.

## Author contributions

LZ developed the prediction method, designed and implemented the experiments, analyzed the result, and wrote the paper. XC conceived the project, designed the experiments, analyzed the result, revised the paper, and supervised the project. N-NG prepared the data, analyzed the result, and revised the paper. HL and J-QL analyzed the result and revised the paper.

### Conflict of interest statement

The authors declare that the research was conducted in the absence of any commercial or financial relationships that could be construed as a potential conflict of interest. The reviewers YW and CW and the handling Editor declared their shared affiliation.

## References

[B1] Adomavicius G. Tuzhilin A. (2005). Toward the next generation of recommender systems: a survey of the state-of-the-art and possible extensions. IEEE Trans. Knowl. Data Eng. 17, 734–749. 10.1109/TKDE.2005.99

[B2] Ammad-ud-din M. Georgii E. Gonen M. Laitinen T. Kallioniemi O. Wennerberg K.. (2014). Integrative and personalized QSAR analysis in cancer by kernelized Bayesian matrix factorization. J. Chem. Inf. Model. 54, 2347–2359. 10.1021/ci500152b 25046554

[B3] Barretina J. Caponigro G. Stransky N. Venkatesan K. Margolin A. A. Kim S.. (2012). The Cancer Cell Line Encyclopedia enables predictive modelling of anticancer drug sensitivity. Nature 483, 603–607. 10.1038/nature11003 22460905 PMC3320027

[B4] Boyd M. R. Paull K. D. (1995). Some practical considerations and applications of the national cancer institute *in vitro* anticancer drug discovery screen. Drug Dev. Res. 34, 91–109. 10.1002/ddr.430340203

[B5] Breese J. S. Heckerman D. Kadie C. (1998). Empirical analysis of predictive algorithms for collaborative filtering, in Proceedings of the Fourteenth conference on Uncertainty in artificial intelligence (Madison: Morgan Kaufmann Publishers Inc), 43–52.

[B6] Cai Y. Tan X. Liu J. Shen Y. Wu D. Ren M.. (2014). Inhibition of PI3K/Akt/mTOR signaling pathway enhances the sensitivity of the SKOV3/DDP ovarian cancer cell line to cisplatin *in vitro*. Chin. J. Cancer Res. 26, 564. 10.3978/j.issn.1000-9604.2014.08.20 25400422 PMC4220257

[B7] Chen J. Cheng G. H. Chen L. P. Pang T. Y. Wang X. L. (2013). Prediction of chemotherapeutic response in unresectable non-small-cell lung cancer (NSCLC) patients by 3-(4,5-dimethylthiazol-2-yl)-5-(3-carboxymethoxyphenyl)-2- (4-sulfophenyl)-2H-tetrazolium (MTS) assay. Asian Pac. J. Cancer Prev. 14, 3057–3062. 10.7314/APJCP.2013.14.5.3057 23803079

[B8] Chen X. Liu M.-X. Yan G.-Y. (2012). Drug–target interaction prediction by random walk on the heterogeneous network. Mol. Biosyst. 8, 1970–1978. 10.1039/c2mb00002d 22538619

[B9] Cortes-Ciriano I. van Westen G. J. Bouvier G. Nilges M. Overington J. P. Bender A.. (2016). Improved large-scale prediction of growth inhibition patterns using the NCI60 cancer cell line panel. Bioinformatics 32, 85–95. 10.1093/bioinformatics/btv529 26351271 PMC4681992

[B10] Costello J. C. Heiser L. M. Georgii E. Gonen M. Menden M. P. Wang N. J.. (2014). A community effort to assess and improve drug sensitivity prediction algorithms. Nat. Biotechnol. 32, 1202–1212. 10.1038/nbt.2877 24880487 PMC4547623

[B11] Daemen A. Griffith O. L. Heiser L. M. Wang N. J. Enache O. M. Sanborn Z.. (2013). Modeling precision treatment of breast cancer. Genome Biol. 14:R110. 10.1186/gb-2013-14-10-r110 24176112 PMC3937590

[B12] Garnett M. J. Edelman E. J. Heidorn S. J. Greenman C. D. Dastur A. Lau K. W.. (2012). Systematic identification of genomic markers of drug sensitivity in cancer cells. Nature 483, 570–575. 10.1038/nature11005 22460902 PMC3349233

[B13] Heiser L. M. Sadanandam A. Kuo W. L. Benz S. C. Goldstein T. C. Ng S.. (2012). Subtype and pathway specific responses to anticancer compounds in breast cancer. Proc. Natl. Acad. Sci. U.S.A. 109, 2724–2729. 10.1073/pnas.1018854108 22003129 PMC3286973

[B14] Kim S. Sundaresan V. Zhou L. Kahveci T. (2016). Integrating domain specific knowledge and network analysis to predict drug sensitivity of cancer cell lines. PLoS ONE 11:e0162173. 10.1371/journal.pone.0162173 27607242 PMC5015856

[B15] Koren Y. (2010). Factor in the neighbors: scalable and accurate collaborative filtering. ACM Trans. Knowl. Discov. Data 4:24 10.1145/1644873.1644874

[B16] Lemire D. (2005). Scale and translation invariant collaborative filtering systems. Inf. Retr. Boston. 8, 129–150. 10.1023/B:INRT.0000048492.50961.a6

[B17] Menden M. P. Iorio F. Garnett M. McDermott U. Benes C. H. Ballester P. J.. (2013). Machine learning prediction of cancer cell sensitivity to drugs based on genomic and chemical properties. PLoS ONE 8:e61318. 10.1371/journal.pone.0061318 23646105 PMC3640019

[B18] Petrelli F. Ghidini M. Lonati V. Tomasello G. Borgonovo K. Ghilardi M.. (2017). The efficacy of lapatinib and capecitabine in HER-2 positive breast cancer with brain metastases: A systematic review and pooled analysis. Eur. J. Cancer 84, 141–148. 10.1016/j.ejca.2017.07.024 28810186

[B19] Riddick G. Song H. Ahn S. Walling J. Borges-Rivera D. Zhang W.. (2011). Predicting *in vitro* drug sensitivity using Random Forests. Bioinformatics 27, 220–224. 10.1093/bioinformatics/btq628 21134890 PMC3018816

[B20] Shoemaker R. H. (2006). The NCI60 human tumour cell line anticancer drug screen. Nat. Rev. Cancer 6, 813–823. 10.1038/nrc1951 16990858

[B21] Shoemaker R. H. Monks A. Alley M. C. Scudiero D. A. Fine D. L. McLemore T. L.. (1988). Development of human tumor cell line panels for use in disease-oriented drug screening. Prog. Clin. Biol. Res. 276, 265–286. 3051021

[B22] Solit D. B. Garraway L. A. Pratilas C. A. Sawai A. Getz G. Basso A.. (2006). BRAF mutation predicts sensitivity to MEK inhibition. Nature 439, 358–362. 10.1038/nature04304 16273091 PMC3306236

[B23] Stanfield Z. Coşkun M. Koyutürk M. (2017). Drug response prediction as a link prediction problem. Sci. Rep. 7:40321 10.1145/3107411.3107459 28067293 PMC5220354

[B24] Staunton J. E. Slonim D. K. Coller H. A. Tamayo P. Angelo M. J. Park J.. (2001). Chemosensitivity prediction by transcriptional profiling. Proc. Natl. Acad. Sci. U.S.A. 98, 10787–10792. 10.1073/pnas.191368598 11553813 PMC58553

[B25] Wang L. Li X. Zhang L. Gao Q. (2017). Improved anticancer drug response prediction in cell lines using matrix factorization with similarity regularization. BMC Cancer 17:513. 10.1186/s12885-017-3500-5 28768489 PMC5541434

[B26] Weinstein J. N. Myers T. G. O'Connor P. M. Friend S. H. Fornace A. J. Jr. Kohn K. W.. (1997). An information-intensive approach to the molecular pharmacology of cancer. Science 275, 343–349. 8994024 10.1126/science.275.5298.343

[B27] Yang W. Soares J. Greninger P. Edelman E. J. Lightfoot H. Forbes S.. (2013). Genomics of Drug Sensitivity in Cancer (GDSC): a resource for therapeutic biomarker discovery in cancer cells. Nucleic Acids Res. 41, D955–D961. 10.1093/nar/gks1111 23180760 PMC3531057

[B28] Yuan H. Paskov I. Paskov H. González A. J. Leslie C. S. (2016). Multitask learning improves prediction of cancer drug sensitivity. Sci. Rep. 6:31619. 10.1038/srep31619 27550087 PMC4994023

[B29] Zhang F. Wang M. Xi J. Yang J. Li A. (2018). A novel heterogeneous network-based method for drug response prediction in cancer cell lines. Sci. Rep. 8:3355. 10.1038/s41598-018-21622-4 29463808 PMC5820329

[B30] Zhang N. Wang H. Fang Y. Wang J. Zheng X. Liu X. S. (2015). Predicting anticancer drug responses using a dual-layer integrated cell line-drug network model. PLoS Comput. Biol. 11:e1004498. 10.1371/journal.pcbi.1004498 26418249 PMC4587957

[B31] Zhao M. Howard E. W. Parris A. B. Guo Z. Zhao Q. Ma Z.. (2017). Activation of cancerous inhibitor of PP2A (CIP2A) contributes to lapatinib resistance through induction of CIP2A-Akt feedback loop in ErbB2-positive breast cancer cells. Oncotarget 8, 58847–58864. 10.18632/oncotarget.19375 28938602 PMC5601698

